# Highly sensitive measurements of disease progression in rare disorders: Developing and validating a multimodal model of retinal degeneration in Stargardt disease

**DOI:** 10.1371/journal.pone.0174020

**Published:** 2017-03-29

**Authors:** Stanley Lambertus, Nathalie M. Bax, Ana Fakin, Joannes M. M. Groenewoud, B. Jeroen Klevering, Anthony T. Moore, Michel Michaelides, Andrew R. Webster, Gert Jan van der Wilt, Carel B. Hoyng

**Affiliations:** 1 Department of Ophthalmology, Donders Institute for Brain, Cognition and Behaviour, Radboud university medical center, Nijmegen, The Netherlands; 2 Moorfields Eye Hospital and UCL Institute of Ophthalmology, London, United Kingdom; 3 Department for Health Evidence, Donders Institute for Brain, Cognition and Behaviour, Radboud university medical center, Nijmegen, The Netherlands; Medizinische Universitat Graz, AUSTRIA

## Abstract

**Background:**

Each inherited retinal disorder is rare, but together, they affect millions of people worldwide. No treatment is currently available for these blinding diseases, but promising new options—including gene therapy—are emerging. Arguably, the most prevalent retinal dystrophy is Stargardt disease. In each case, the specific combination of *ABCA4* variants (> 900 identified to date) and modifying factors is virtually unique. It accounts for the vast phenotypic heterogeneity including variable rates of functional and structural progression, thereby potentially limiting the ability of phase I/II clinical trials to assess efficacy of novel therapies with few patients. To accommodate this problem, we developed and validated a sensitive and reliable composite clinical trial endpoint for disease progression based on structural measurements of retinal degeneration.

**Methods and findings:**

We used longitudinal data from early-onset Stargardt patients from the Netherlands (development cohort, *n* = 14) and the United Kingdom (external validation cohort, *n* = 18). The composite endpoint was derived from best-corrected visual acuity, fundus autofluorescence, and spectral-domain optical coherence tomography. Weighting optimization techniques excluded visual acuity from the composite endpoint. After optimization, the endpoint outperformed each univariable outcome, and showed an average progression of 0.41° retinal eccentricity per year (95% confidence interval, 0.30–0.52). Comparing with actual longitudinal values, the model accurately predicted progression (*R*^*2*^, 0.904). These properties were largely preserved in the validation cohort (0.43°/year [0.33–0.53]; prediction: *R*^*2*^, 0.872). We subsequently ran a two-year trial simulation with the composite endpoint, which detected a 25% decrease in disease progression with 80% statistical power using only 14 patients.

**Conclusions:**

These results suggest that a multimodal endpoint, reflecting structural macular changes, provides a sensitive measurement of disease progression in Stargardt disease. It can be very useful in the evaluation of novel therapeutic modalities in rare disorders.

## Introduction

Inherited blindness affects millions of people worldwide—the majority suffering from retinal disease [1]. Inherited retinal disorders now represent the primary cause of blindness in the working age population in the UK, and secondary in childhood [2]. They are clinically and genetically heterogeneous, caused by sequence variants in more than 300 distinct genes (RetNet) http://www.sph.uth.tmc.edu)/. Mutations in the ATP-binding cassette, subfamily A, member 4 (*ABCA4)* gene are linked to arguably the most common retinal dystrophy: autosomal recessive Stargardt disease (STGD1) [3]. Each case of STGD1 is, in a sense, unique by specific combinations of pathogenic *ABCA4* variants (> 900 variants identified to date) and modifying factors. Consequently, the natural course is highly variable, ranging from severe early-onset rapid degeneration [4, 5] to relatively mild late-onset disease [6, 7]. The eventual vision loss results from progressive impairment and degeneration of photoreceptors and their supporting retinal pigment epithelium (RPE) [8].

Recently, significant advancement has been made in the development of therapies that aim to slow disease progression, or even to restore lost photoreceptors in STGD1. These include replacement of *ABCA4* by gene therapy [9], cell-based therapies [10], and pharmacological strategies including slowing the visual cycle or inhibition of vitamin A dimerization [11, 12]. Clinical trials are currently recruiting patients to assess safety and efficacy. However, successful evaluation of these therapies critically hinges on sensitive and reliable measures for disease progression.

To monitor efficacy of a treatment, current trials generally use functional endpoints (S1 Table). One of the most widely used U.S. Food and Drug Administration-approved endpoints is best-corrected visual acuity (BCVA) [13]. However, the main disadvantage of BCVA lies in its extremely variable deterioration rate in patients with retinal dystrophies. Moreover, visual acuity decline can be a late phenomenon following a long period of pathophysiological changes [14]. As a result, the endpoint has an unfavorable signal-to-noise ratio, which leads to a need for longer follow-up and large cohorts. This setup is impossible to achieve given the unacceptable long time frame and the rarity of these disorders.

However, studies suggest that structural abnormalities gradually expand centrifugally [5, 15], starting from the foveal center towards the periphery. Although loss of foveal function is highly important from a patient’s perspective, it is only one step in the overall pattern of retinal degeneration. This pattern is thought to initiate with melanization abnormalities in the RPE [16, 17], and is trailed by changes in lipofuscin fluorophores [18–20], degeneration of the RPE [21–23], and loss of the ellipsoid zone (EZ) and external limiting membrane (ELM) [24–27]. However, these transition zones are not present in every case, and do not always start at the center of the macula; many are still not well understood.

A composite outcome measure is likely to outperform single candidate outcome measures [28] in accurately measuring short-term progression, and can therefore increase statistical power of pivotal clinical trials. In this study, we chose to measure four structural metrics of expanding transition zones and one psychophysical metric over time in a cohort of patients with early-onset STGD1. These included questionably and definitely decreased autofluorescence (QDAF and DDAF), and loss of the EZ, ELM and BCVA. Next, we assessed intra- and inter-grader differences. Having standardized all metrics, we then calculated an optimal weighted composite. The same measurements were made in a second patient cohort, and, using the composite, we predicted and compared progression with real longitudinal values. We subsequently ran a simulation to examine the power of the composite endpoint and alternatives to detect a difference in outcome given a theoretical intervention with a significant impact on progression rate.

## Results

### Characteristics of the initial patient population

A cohort of 14 patients with early-onset STGD1 ascertained at the Radboud university medical center in Nijmegen had a median age at disease onset of 9 years (range, 4–11). Seventy-four eye-visits of 28 eyes were included in this study (range, 2–4 visits per eye). At the time of inclusion, all eyes had abnormal fundus autofluorescence (FAF) imaging and evidence of loss of photoreceptors on spectral-domain optical coherence tomography (SD-OCT), primarily limited to the macula at their baseline visit. Due to their early disease onset and rapidly progressive macular changes, a significant proportion of patients were expected to progress to retina-wide disease over time [4, 5, 29]. The follow-up time ranged from 1.1 to 9.7 years (median, 4.7).

### Visual acuity measurements indicate extra-macular dysfunction

**Fig 1:**
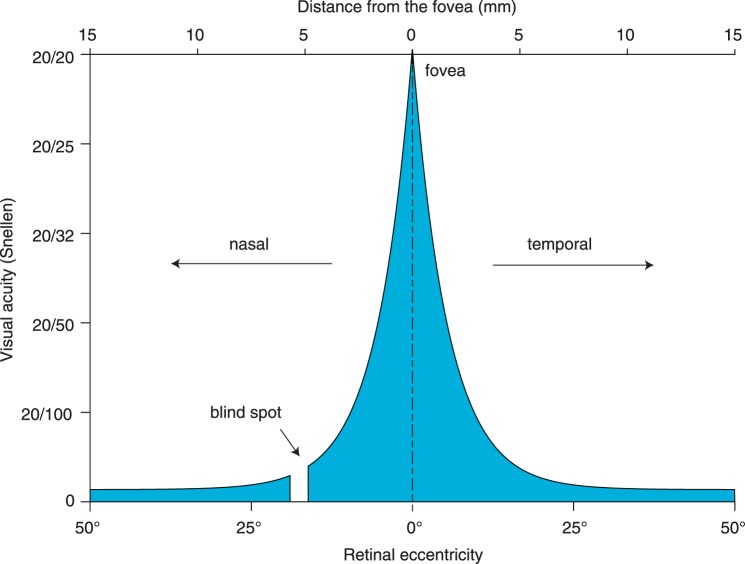
Visual acuity as a function of degrees of retinal eccentricity.

At the first visit, the Nijmegen cohort had a median BCVA of 20/205 Snellen in both eyes. The highest BCVA can be obtained at the fovea and diminishes rapidly by retinal eccentricity (*ε*) as shown in Fig 1 [30]. According to Fig 1, 20/205 Snellen corresponds to 12.76° of *ε* by BCVA (*ε*_*BCVA*_), the equivalent diameter being 7.7 mm. Assuming a 5.5 mm diameter of the macula, the high degree of eccentricity indicated that the patients’ visual function corresponded to extra-macular disease at baseline.

### Structural parameters are measured with high reproducibility

To assess the accuracy and reliability of measurements on FAF imaging and SD-OCT, we analyzed inter- and intra-grader agreements. These included transition zones of QDAF and DDAF areas, and transverse loss of the EZ and ELM. The absolute mean (± standard deviation) differences within one grader for *ε*_*QDAF*_, *ε*_*DDAF*_, *ε*_*EZ*_ and *ε*_*ELM*_ were 0.19 ± 0.18°, 0.20 ± 0.23°, 0.10 ± 0.12°, and 0.20 ± 0.22°, respectively. The intra-grader measurements were highly correlated with intraclass correlation coefficients (95% confidence interval) of 0.995 (0.989–0.997), 0.994 (0.986–0.997), 0.998 (0.993–0.999), and 0.998 (0.994–0.999), respectively. The absolute mean differences between graders were 0.26 ± 0.23°, 0.22 ± 0.21°, 0.26 ± 0.23°, and 0.23 ± 0.18°, respectively. The measurements from both graders were highly correlated with intraclass correlation coefficients of 0.992 (0.986–0.995), 0.994 (0.990–0.996), 0.992 (0.971–0.998), and 0.997 (0.992–0.999), respectively.

### Initial transition zones on retinal imaging

**Fig 2:**
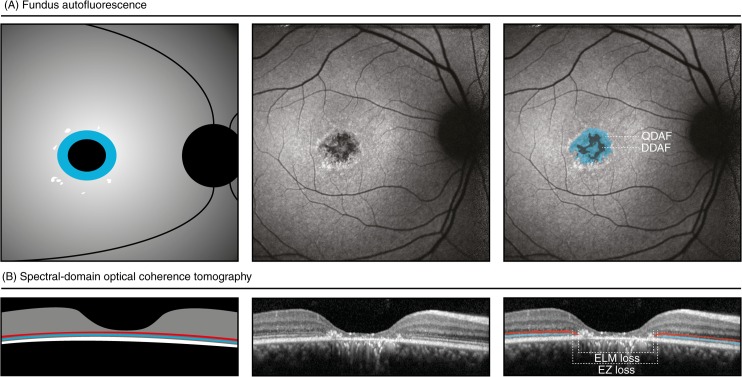
Schematic and representative images of measurements in fundus autofluorescence imaging and spectral-domain optical coherence tomography. Patient 9: area of questionably decreased autofluorescence (QDAF, blue), 1.37 mm^2^; area of definitely decreased autofluorescence (black), 0.33 mm^2^; transverse loss of external limiting membrane (ELM-loss, red), 1.75 mm, transverse loss of ellipsoid zone (EZ-loss, blue), 2.24 mm; best-corrected visual acuity, 20/100; *ABCA4* variants, c.1622T>C;3113C>T:p.[Leu541Pro;Ala1038Val] and c.6316C>T:p.(Arg2106Cys).

We measured the abnormalities of lipofuscin fluorophores in the RPE by determining both the transition zones of DDAF and QDAF on FAF imaging as shown in Fig 2A [20, 31]. At the first visit, the median *ε*_*DDAF*_ and *ε*_*QDAF*_ for both eyes were 1.80° (range, 0–5.60) and 4.22° (range, 1.68–7.67), equivalent to an area of 0.92 mm^2^ and 5.03 mm^2^, respectively. Additionally, we measured the horizontal loss of the photoreceptor-related ELM and EZ through the foveal center on SD-OCT (Fig 2B). At the first visit, the median *ε*_*ELM*_ and *ε*_*EZ*_ for both eyes were 5.09° (range, 2.50–10.71) and 3.54° (range, 2.99–7.83), equivalent to a loss of 3.05 mm and 2.12 mm, respectively.

### A structural composite measure of expanding transition zones outperforms univariable measures

**Fig 3:**
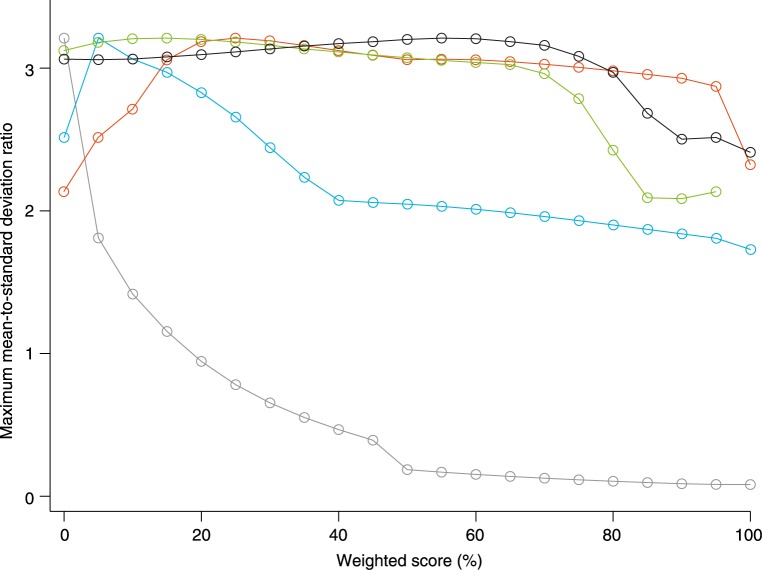
Highest potential mean-to-standard deviation ratio (MSDR) for each single outcome measure at different weightings with all possible weight combinations of the other metrics. MSDRs for best-corrected visual acuity (grey) decrease at increasing weight. MSDRs for transition zones of questionably decreased autofluorescence (blue) increase until 25% weight, but gradually decrease at higher weights. MSDRs for transition zones of definitely decreased autofluorescence decrease at weights higher than 5%. MSDRs for loss of the ellipsoid zone (green) are constant, but decrease substantially at weights higher than approximately 70%. MSDRs for loss of the external limiting membrane (black) decrease at weights higher than approximately 80%.

**Fig 4:**
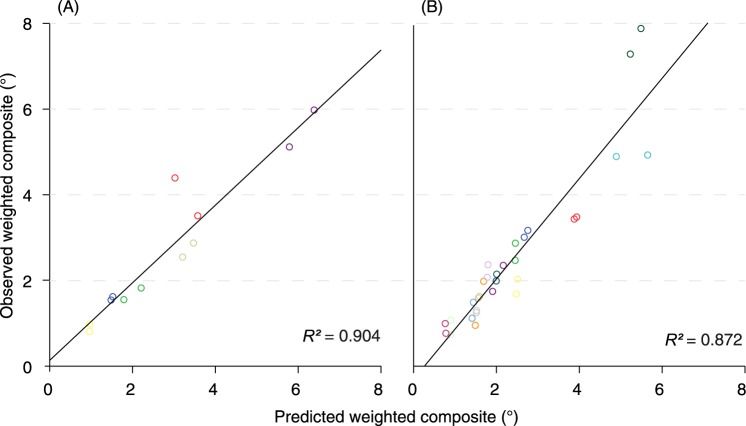
Weighted composite score and predicted outcome. Matching colors represent the right and left eye of the same patient. (A) Results from six early-onset Stargardt patients. (B) The predicted outcome in the replication cohort showed comparable results.

We assessed the change of each individual parameter over time by linear mixed-effects models. The models accounted for between-patients and between-eyes effects. We then calculated the sensitivity of each parameter by the ratio of the population mean slope, i.e., overall disease progression, and the residual standard deviation (mean-to-standard-deviation ratio, MSDR). *ε*_*QDAF*_ had the highest sensitivity (MSDR, 2.32), whereas *ε*_*BCVA*_ had the lowest (MSDR, 0.08). The MSDR for *ε*_*EZ*_ could not be obtained as there were not sufficient measurements available. Sensitivities of all individual parameters are shown in Table 1. We constructed a composite variable from changes in *ε* as measured by BCVA, QDAF, DDAF, EZ, and ELM. Results from MSDR calculations of every potential weighting combination indicated that the most sensitive composite consisted of a weighted mean of changes in *ε*_*BCVA*_ (0%), *ε*_*QDAF*_ (25%), *ε*_*DDAF*_ (5%), *ε*_*EZ*_ (55%), and *ε*_*ELM*_ (15%). We observed an overall progression rate of 0.41°/year (95% confidence interval, 0.30–0.52). Potential MSDRs for all measures with different weighting scores are shown in Fig 3. Based on the weighted composite score we predicted changes in *ε* of six patients with a third or fourth visit. The predicted values correlated strongly with the measured weighted composite score (*R*^*2*^, 0.904; slope, 0.90 [0.70–1.11]; intercept, 0.14 [-0.55–0.83]; Fig 4A).

**Table 1:** Yearly progression rate of changes in retinal eccentricity (*ε*) by visual function, fundus autofluorescence and optical coherence tomography.

	Slope (mean)	Residual (SD)	MSDR
Univariable outcomes			
*ε*_*BCVA*_	0.31°/year	3.77°/year	0.08
*ε*_*QDAF*_	0.32°/year	0.14°/year	2.32
*ε*_*DDAF*_	0.58°/year	0.33°/year	1.73
*ε*_*ELM*_	0.34°/year	0.17°/year	1.97
*ε*_EZ_*	0.38°/year	*	*
Composite outcomes			
Unweighted (*ε*_*BCVA*_, *ε*_*QDAF*_, *ε*_*DDAF*_, *ε*_*ELM*_, *ε*_*EZ*_)	0.35°/year	1.24°/year	0.28
Unweighted (*ε*_*QDAF*_, *ε*_*DDAF*_, *ε*_*ELM*_, *ε*_*EZ*_)	0.47°/year	0.24°/year	2.00
Optimal weight (15% *ε*_*QDAF*_, 5% *ε*_*DDAF*_, 15% *ε*_*ELM*_, 55% *ε*_*EZ*_)	0.41°/year	0.13°/year	3.21

BCVA = best-corrected visual acuity, QDAF = questionably decreased autofluorescence, DDAF = definitely decreased autofluorescence, ELM = external limiting membrane, EZ = ellipsoid zone, MSDR = mean-to standard deviation ratio, SD = standard deviation, *ε* = retinal eccentricity.

*There were limited measurements available because it exceeded retinal scans.

### Accurate prediction of disease progression is validated in a replication cohort

Using the identical weighted score in the mixed-effects model, we predicted the progression in a separate replication cohort of 18 Stargardt patients, ascertained at Moorfields Eye Hospital in London. One hundred and thirty-eight eye-visits were included (range, 2–6 visits per patient). The London cohort was not significantly different to the Nijmegen cohort: with a median age at disease onset of 8 years (range, 5–11; Mann-Whitney U, *P* = 0.419), and follow-up from 1.0 to 11.0 years (median, 4.5; Mann-Whitney U, *P* = 0.722). We observed an overall progression of 0.43°/year (95% confidence interval, 0.33–0.53). Cohort characteristics compared to the Nijmegen cohort are further described in Table 2. Predicted values correlated with the measured weighted composite score (*R*^*2*^, 0.872; slope, 1.17 [0.99–1.34]; intercept, -0.29 [-0.77–0.18]; Fig 4B).

**Table 2:** Characteristics of early-onset Stargardt cohorts from Radboud university medical center (Radboudumc) and Moorfields Eye Hospital (MEH).

	Radboudumc	MEH
Patients	7 men	11 men
	7 women	7 women
Baseline characteristics		
Age at onset (years)	9 (4–11)	8 (5–11)
Age at baseline (years)	13 (9–26)	14 (8–25)
*ε*_*BCVA*_ (°, Snellen equivalent)	12.76 (9.85–24.00), 20/200	12.62 (6.31–18.94), 20/200
*ε*_*QDAF*_ (°, area equivalent)	4.22 (1.68–7.67), 5.03 mm^2^	4.64 (1.44–9.98), 6.10 mm^2^
*ε*_*DDAF*_ (°, area equivalent)	1.80 (0–5.60), 0.92 mm^2^	2.40 (0–6.41), 1.63 mm^2^
*ε*_*ELM*_ (°, transverse equivalent)	5.09 (2.50–10.71), 3.05 mm	4.82 (2.06–7.71), 2.89 mm
*ε*_*EZ*_ (°, transverse equivalent)	3.54 (2.99–7.83), 2.12 mm	5.87 (3.86–8.58), 3.52 mm
Disease progression		
Follow-up (years)	4.73 (1.13–9.71)	4.47 (1.0–10.99)
Progression (°/year)	0.41 (95% CI, 0.30–0.52)	0.43 (95% CI, 0.33–0.53)

Median and range are shown for baseline characteristics and follow-up. BCVA = best-corrected visual acuity, CI = confidence interval, QDAF = questionably decreased autofluorescence, DDAF = definitely decreased autofluorescence, *ε* = retinal eccentricity, ELM = external limiting membrane, EZ = ellipsoid zone, SE = standard error, *ε* = retinal eccentricity.

### Simulation reveals high statistical power despite small numbers of patients and short follow-up

**Fig 5:**
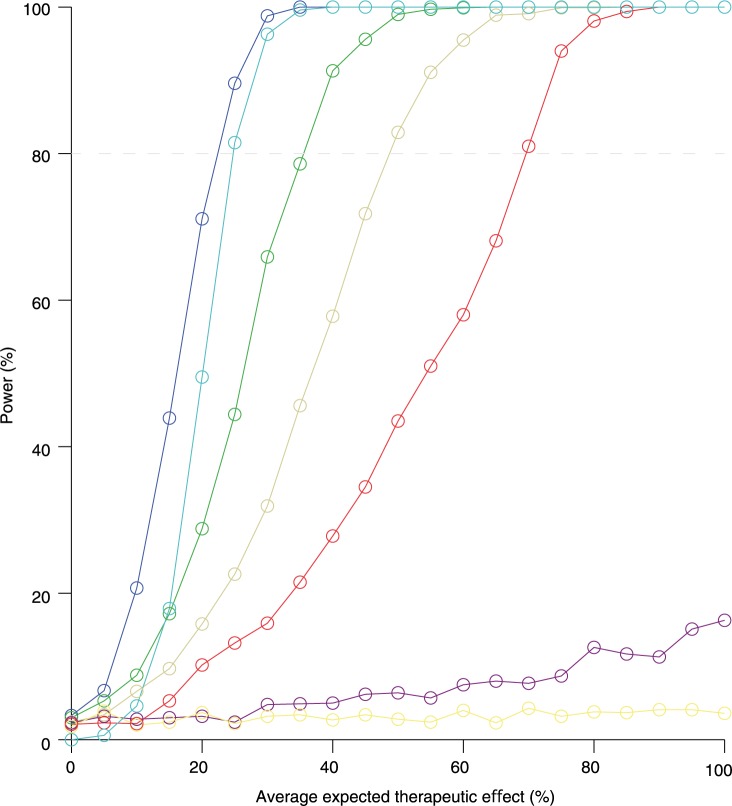
Power calculations of a simulated therapeutic trial based on fourteen early-onset Stargardt patients. Dark blue line: a power of 80% is reached with a 25% overall treatment effect and a two-year follow-up period. Purple line: the power will drastically decrease when best-corrected visual acuity is included in the outcome measure. Turquoise line: worse eye treated. Green line: one-year follow-up. Ocher line: unweighted structural composite. Red line: non-paired trial design. Yellow line: best-corrected visual acuity as a single outcome measure.

Finally, to assess the value of the composite biomarker in an interventional trial, we simulated a randomized-controlled paired trial in 14 patients, with a two-year follow-up period, and different treatment effects (Fig 5). Using the optimized weighted composite score as the primary endpoint, we obtained a statistical power of > 80% (with a significance level of 0.05) in the case of a 25% treatment effect. Unweighted structural scores decreased the power by approximately 50%.

## Discussion

The genotypic and phenotypic heterogeneity of rare diseases are a challenge for designing therapeutic clinical trials using conventional parameters. This affects many patients; current estimates are between 6.5 and 9.9 million inhabitants of the EU28 countries (1.3–2.0%). Jointly, these diseases represent a relevant public health issue [32], and to evaluate novel treatments, better strategies are needed. Current strategies use biomarkers, which are often insufficient to provide appropriate sample size calculations, or require long-term follow-up (S1 Table). However, an integrated approach of these individual biomarkers can result in a reliable and sensitive marker for disease progression. In this paper, we showed that such markers can be developed using composite endpoints and weighting optimization techniques.

The composite endpoint that we developed to sensitively measure disease progression in patients with early-onset STGD1 was based on its spatiotemporal disease course. Although the exact pathophysiological mechanisms of the disease pattern are still not completely understood, natural history studies suggest a centrifugal expansion from macula-only to potentially retina-wide disease [5, 15, 16, 29, 33]. Visual acuity failed to detect this gradual expansion due to its low signal-to-noise ratio in the composite model. In the final model, the expansion of different transition zones is analyzed by two widely available imaging techniques. SD-OCT can visualize photoreceptor damage, represented by loss of the ellipsoid zone, followed by loss of the external limiting membrane [34–36], and FAF imaging can detect RPE atrophy associated with photoreceptor dysfunction/loss [26]. In the future, the composite model may be further optimized by incorporating more sophisticated retinal imaging techniques such as adaptive optics scanning light ophthalmoscopy which affords *in-vivo* cellular imaging. Parameters derived from electrophysiological assessment could also potentially strengthen the composite model, although it is limited by significantly greater test-retest variability than aforementioned structural testing [37–39].

We are aware that, ultimately, the value of novel therapeutic modalities should be inferred from their impact on outcomes that matter to patients. In the context of eye disease, these would certainly include vision and the impact that visual impairments have on daily life (patient-reported outcome measures). In addition, the long-term safety of such novel treatments should be safeguarded. For these purposes, the metric that we have developed in this study is unlikely to be appropriate in isolation, since its focus lies on structural abnormalities alone. We believe its value mainly derives from the ability to rationally select promising novel treatment modalities and also potentially facilitate patient selection for recruitment to clinical trials. Consequently, it will need to be demonstrated that the employed structural parameters correlate with functional outcome in the long term.

Multimodal analysis is a powerful technique, potentially reducing costs and duration of clinical trials and also likely reducing beta errors in data analysis, thereby hopefully facilitating effective treatments being identified more readily and rapidly for patients with rare diseases. There is also the possibility of further improvements with the inclusion of other biomarkers in the future, and the potential to be extrapolated to other disorders.

## Materials and methods

### Patient selection

We selected patients from the Stargardt databases of the Departments of Ophthalmology at Radboud university medical center (Nijmegen, The Netherlands) and Moorfields Eye Hospital (London, United Kingdom). We used the patient data from Radboud university medical center to develop the progression model, and the data from Moorfields Eye Hospital to replicate the study. We included patients with the faster progressive early-onset form of STGD1, harboring two or more likely disease-causing sequence variants in *ABCA4* (Table 3), and with at least one year follow-up with FAF imaging and/or SD-OCT The early-onset phenotype typically presents with foveal atrophy that may precede the development of yellow-white fundus flecks. Early-onset STGD1 is associated with the most rapid deterioration of all patients with STGD1 [5]. We only included patients with a reported disease onset <12 years of age [4, 5]. We excluded (1) patients with very early disease in which only thickening of the external limiting membrane was present, because this would preclude the OCT measurements, (2) patients with advanced disease characterized by RPE atrophy beyond the vascular arcades at first presentation, and (3) patients who participated in an interventional trial [40]. This study was approved by the local ethics committee on Research Involving Human Subjects of the Radboud university medical center “Commissie Mensgebonden Onderzoek Regio Arnhem-Nijmegen” and the National Research Ethics Service (NRES) Committee London—Camden & Islington, and was performed in accordance with the Declaration of Helsinki. All patients provided informed consent prior to receiving additional ophthalmologic examinations. Written informed consent was obtained from parents, caretakers, or guardians on behalf of the children below 18 years (at Radboud university medical center) or below 16 years (at Moorfields Eye Hospital). Additional written informed consent was obtained from children between 12 and 17 years old (at Radboud university medical center) or between 6 and 15 years old (not necessary, but preferable at Moorfields Eye Hospital).

**Table 3:** *ABCA4* variants in early-onset Stargardt patients from Radboud university medical center (Radboudumc) and Moorfields Eye Hospital (MEH).

**Radboudumc**				
Patient	Variant 1		Variant 2	
1	c.5461-10T>C	p.[Thr1821Valfs*13,Thr1821Aspfs*6]	c.5461-10T>C	p.[Thr1821Valfs*13,Thr1821Aspfs*6]
2	c.5461-10T>C	p.[Thr1821Valfs*13,Thr1821Aspfs*6]	c.214G>A	p.(Gly72Arg)
3	c.5461-10T>C	p.[Thr1821Valfs*13,Thr1821Aspfs*6]	c.5537T>C	p.(Ile1846Thr)
4	c.768G>T	p.(?)	c.1822T>A	p.(Phe608Ile)
5	c.3033-?_3364+?del	p.(?)	c.5714+5G>A	p.(?)
6	c.5461-10T>C	p.[Thr1821Valfs*13,Thr1821Aspfs*6]	c.5337C>A	p.(Tyr1779*)
7	c.286A>G	p.(Asn96Asp)	c.286A>G	p.(Asn96Asp)
8	c.5461-10T>C	p.[Thr1821Valfs*13,Thr1821Aspfs*6]	c.4773+1G>A	p.(?)
9	c.1622T>C;3113C>T	p.[Leu541Pro;Ala1038Val]	c.6316C>T	p.(Arg2106Cys)
10	c.768G>T	p.(?)	c.768G>T	p.(?)
11	c.3033-?_3364+?del	p.(?)	c.5714+5G>A	p.(?)
12	c.4128+1G>A	p.(?)	c.3259G>A	p.(Glu1087Lys)
13	c.4128+1G>A	p.(?)	c.3259G>A	p.(Glu1087Lys)
14	c.4139C>T	p.(Pro1380Leu)	c.2160+1G>T	p.(?)
**MEH**				
Patient	Variant 1		Variant 2	
1	c.3191-1G>T	p.(?)	c.4139C>T	p.(Pro1380Leu)
2	c.4462T>C	p.(Cys1488Arg)	c.4462T>C	p.(Cys1488Arg)
3	c.6079C>T	p.(Leu2027Phe)	c.3322C>T	p.(Arg1108Cys)
4	c.6479+1G>A	p.(?)	c.6479+1G>A	p.(?)
5	c.6479+1G>A	p.(?)	c.6479+1G>A	p.(?)
6	c.4469G>A	p.(Cys1490Tyr)	c.3197T>G	p.(Met1066Arg)
7	c.4253+4C>T	p.(?)	c.4253+4C>T	p.(?)
8	c.5461-10T>C	p.[Thr1821Valfs*13,Thr1821Aspfs*6]	c.3299T>A	p.(Ile1100Asn)
9	c.768G>T	p.(?)	c.4139C>T	p.(Pro1380Leu)
10	c.3081T>G	p.(Tyr1027*)	c.3081T>G	p.(Tyr1027*)
11	c.6286G>A	p.(Glu2096Lys)	c.2894A>G	p.(Asn965Ser)
12	c.4577C>T	p.(Thr1526Met)	c.3322C>T	p.(Arg1108Cys)
13	c.93G>A	p.(Trp31*)	c.2522A>C	p.(Gln841Pro)
14	c.4139C>T	p.(Pro1380Leu)	c.1957C>T	p.(Arg653Cys)
15	c.6729+4_6729+18del	p.(?)	c.6729+4_6729+18del	p.(?)
	AGTTGGCCCTGGGGC		AGTTGGCCCTGGGGC	
16	c.5714+5G>A	p.(?)	c.1622T>C;3113C>T	p.[Leu541Pro;Ala1038Val]
17	c.6729+4_6729+18del	p.(?)	c.6729+4_6729+18del	p.(?)
	AGTTGGCCCTGGGGC		AGTTGGCCCTGGGGC	
18	c.2912C>A	p.(Thr971Asn)	c.2912C>A	p.(Thr971Asn)

del = deletion, fs = frame shift, ins = insertion

* = stop codon.

### Clinical examinations

We reviewed the records and imaging databases to extract information including BCVA, FAF imaging, and SD-OCT. Best-corrected visual acuity was measured using a Snellen or Early Treatment Diabetic Retinopathy Study chart. Short-wave FAF imaging (λ = 488 nm, emission 500–700 nm) was performed using a confocal scanning laser ophthalmoscope (Spectralis HRA+OCT or HRA2, Heidelberg Engineering, Heidelberg, Germany). The field of view was set at 30°×30° or 55°×55° and was centered at the macula. Cross-sectional images were obtained using SD-OCT (Spectralis HRA+OCT, Heidelberg Engineering, Heidelberg, Germany) centered at the macula (S1 Dataset).

### Functional measurements

We analyzed data with SAS Statistical Analysis Software Version 9.2 (SAS Institute, Cary, NC). Best-corrected visual acuity can be expressed in retinal eccentricity (*ε*) to estimate the spatial extent of retinal dysfunction spatially. It has been calculated previously that an object must grow by 0.2° in size to maintain BCVA for each degree of eccentricity [30]. The BCVA is therefore reduced by a factor of 1/1.2 for each degree. This results in a transformed BCVA to the estimated equivalent of *ε*:

$$BCVA={\left(\frac{1}{1.2}\right)}^{\varepsilon }$$ (1)

which can be written as

$$\varepsilon_{BCVA}={log}_{1/1.2}\;BCVA$$ (2)

### Quantitative measurements on imaging

Abnormalities that were expected to consistently increase over time were included for quantification. Two independent authors (S.L. and N.M.B.), blinded to the each other’s findings, manually delineated areas of abnormal autofluorescence signals based on darkness levels on FAF imaging, and the loss of retinal layers on SD-OCT. These included areas of questionably decreased autofluorescence (QDAF), definitely decreased autofluorescence (DDAF), transverse loss of the external limiting membrane (ELM) and ellipsoid zone (EZ) on the OCT scan through the fovea. All measurements were standardized to retinal eccentricity. One degree of eccentricity corresponds to approximately 0.3 mm on the retina [41]. Therefore, the eccentricity can be calculated as the radius of the circular equivalents of the sum of QDAF and DDAF areas using the previously reported conversion factor:

$$\varepsilon_{QDAF}={0,3}^{-1}\sqrt{\frac{QDAF}{\pi }}$$ (3)

and

$$\varepsilon_{DDAF}={0,3}^{-1}\sqrt{\frac{DDAF}{\pi }}$$ (4)

As the transverse horizontal loss of retinal layers represents the diameter of a circular equivalent, the eccentricities of ELM loss and EZ loss could be calculated as follows:

$$\varepsilon_{EZ}={0,3}^{-1}\frac{EZ}{2}$$ (5)

and

$$\varepsilon_{ELM}={0,3}^{-1}\;\frac{ELM}{2}$$ (6)

### Reproducibility of measurements

If the discrepancy between graders exceeded 1°, the graders were asked to reach consensus on the location and extent of the transition zone. To assess the intra-grader reproducibility, one of the two graders (S.L.) measured each image by each method twice, with a two-month interval between gradings of the same image. We calculated the absolute inter- and intrarater agreement of *ε*_*QDAF*_, *ε*_*DDAF*_ (5%), *ε*_*ELM*_, and *ε*_*EZ*_ by intraclass correlation coefficients with 95% confidence intervals. Averaged values of the graders were used for final analyses.

### Modelling disease progression

The composite ΔC was constructed from changes in *ε* as measured in all univariable biomarkers:

$${{\Delta C}_{ij}({\Delta t}_{ik})=(a\times {[\varepsilon_{BCVA}}_{ij}(t_{ik})-\varepsilon_{BCVA}}_{ij}(t_{i,0})]+b\times {[\varepsilon_{QDAF}}_{ij}(t_{ik})-{\varepsilon_{QDAF}}_{ij}(t_{i,0})]+c\times {[\varepsilon_{DDAF}}_{ij}(t_{ik})-{\varepsilon_{DDAF}}_{ij}(t_{i,0})]+d\times {[\varepsilon_{EZ}}_{ij}(t_{ik})-{\varepsilon_{EZ}}_{ij}(t_{i,0})]+e\times {[\varepsilon_{ELM}}_{ij}(t_{ik})-{\varepsilon_{ELM}}_{ij}(t_{i,0})])/(a+b+c+d+e)$$ (7)

- a, b, c, d, and e are weighting scores for each biomarker.

The composite biomarker was used to detect disease progression in a linear two-level random effects mixed model, which can describe expansion rates of a transition zone quite well within a short period. It accounts for variations between patients and between the eyes of each patient, and can thus incorporate a potential non-linear process in the variance components of these random effect [42]:

$${\Delta C}_{ij}\left({\Delta t}_{ik}\right)=\left[s+s_{i}+s_{ij}\right]\times {\Delta t}_{ik}+E_{ijk}$$ (8)

- ΔC_ij_(Δt) is the change of the composite score from baseline for the i^th^ patient in eye j at time since baseline Δt,
- Δt_ik_ is the k^th^ follow-up time for patient i,
- s is the mean population slope (first level fixed effect),
- s_i_ are the deviations of the i^th^ patient’s slope from the population value (independent second level random effects),
- s_ij_ are the deviations of the slope of both eyes in patient i from his mean regression line (independent third level random effects),
- E_ijk_ is the residual error.

The intercept of the model was set to zero, because differences from baseline were used.

### Weighting scores optimization

Weighting scores were subsequently chosen by an optimization criterion [28], which was constructed by the ratio of the population mean slope and the residual standard deviation (MSDR). The criterion was empirically evaluated for different combinations of weighting scores of parameters:

$$MSDR=\frac{s}{RMSE}$$ (9)

- s is the mean population slope,
- RMSE (root-mean-square error) is the residual standard deviation, a scale-dependent measure for accuracy.

The total number of unique combinations follows a binomial coefficient (n+a−1n), where *n* is the number of intervals, and *a* is the number of biomarkers. Five biomarkers with 5% intervals (20 steps from 0 to 100%) resulted in $\frac{24!}{20!4!}=10626$ combinations, which could be tested within reasonable computational time. A weighting of zero resulted in exclusion of that particular biomarker. When a certain biomarker was missing or not measurable, the ΔC was calculated by the changes in the other biomarkers with their respective weighting scores. The combination with the highest MSDR was eventually used in the final model (S2 Dataset and S1 Appendix).

### Validation and replication of the model

In a subset of patients (six), in which three or more visits were available, we calculated disease progression of the last visit. These final visits were excluded in the construction of the weighted composite. We compared these scores with the predicted scores based on the mixed model. Using identical weighting scores, we replicated the study in another cohort.

### Trial simulations

Based on these calculations, the optimal composite score difference was used to perform simulations of a two-year interventional trial with the change in retinal eccentricity as primary outcome measured by the composite biomarker. By introducing a potential treatment effect, we calculated the expected power of a trial with these patients. The treated eye was randomly assigned by a Bernoulli distribution. The progression was then simulated as follows:

$$Difference\;of\;{\Delta C}_{ij}({\Delta t}_{il+x})={[\Delta C}_{ij}({\Delta t}_{il+x})\pm adjusted\;RMSE-\delta ]-{\Delta C}_{ij}({\Delta t}_{il})$$ (10)

where

$$\delta =effect\;size\;(\pm 0.1)\times [predicted\;{\Delta C}_{ij}({\Delta t}_{il+x})-predicted\;{\Delta C}_{it}({\Delta t}_{il})]$$ (11)

- difference of ΔC_i,t_(Δt_i,l+x_) is the difference of the change of the composite score from baseline for the ith patient in the j^th^ eye t at x years after the last visit l, compared to the last visit.
- the adjusted RMSE was calculated for residual errors of follow-up measurements without baseline measurements (Δt_i,0_), where E_ij_ is zero in all patients,
- the treatment effect δ was simulated from 0 to 1 by steps of 0.05 in treated eyes.

As the degree of abnormalities are highly correlated between left and right eyes [21, 23] the power can be increased considerably when the fellow eye serves as the paired control. A paired-samples T-test was therefore performed to assess the differences between eyes in progression of changes in retinal eccentricity. Power calculations were performed by 10000 simulations for each data point (S2 Appendix).

## Supporting information

S1 Table Current gene therapy trials for retinal dystrophies, including expected number of patients enrolled, timeframe and endpoints that assess efficacy. (PDF)

S1 Dataset Functional and structural measurements from the development (Radboud university medical center) and external validation (Moorfields Eye Hospital) cohort. (XLSX)

S2 Dataset Outcome of all combinations of weighting scores. (XLSX)

S1 Appendix Syntax for the weighting scores optimization. (PDF)

S2 Appendix Syntax for the trial simulations. (PDF)
