# Supplementary material for: Highly sensitive measurements of disease progression in rare disorders: Developing and validating a multimodal model of retinal degeneration in Stargardt disease
Source: PLoS One. 2017 Mar 29;12(3):e0174020. doi: 10.1371/journal.pone.0174020 (PMC5371327; doi:10.1371/journal.pone.0174020)
Supplement: S1 Table — (PDF) [file pone.0174020.s001.pdf]

S1 Table. Current gene therapy trials for retinal dystrophies, including expected number of patients enrolled, timeframe and endpoints that assess efficacy.

NCT	Phase	Disease	Gene	Patients	Timeframe (months)	Endpoints for efficacy
01494805	I/II	AMD	*sFLT01*	40	36	BCVA, CNV lesion, foveal thickness
01301443	I	AMD	-	21	0,5	OCT
01024998	I	AMD	*sFLT01*	34	12	Decreased retinal thickness
02341807	I/II	CHM	*CHM*	10	24	Visual function
02077361	I	CHM	*CHM*	6	24	Visual field, visual function
01461213	I/II	CHM	*CHM*	14	6	Visual acuity
01496040	I/II	LCA	*RPE65*	9	12	ERG, patient questionnaire, visual acuity, color vision, pupillometry, microperimetry, dark adaptation
00999609	III	LCA	*RPE65*	24	12	Mobility testing
00821340	I	LCA	*RPE65*	10	36	Visual function
00749957	I/II	LCA	*RPE65*	12	24	Visual fields, visual acuity, ERG
00643747	I/II	LCA	*RPE65*	12	12	Visual function
00516477	I	LCA	*RPE65*	12	60	Visual function
00481546	I	LCA	*RPE65*	15	180	Visual function
01482195	I	LCA	*MERTK*	6	12	Visual function
01367444	I/II	STGD1	*ABCA4*	46	12	Delay in retinal degeneration by BCVA, microperimetry, full-field static/kinetic perimetry, OCT and FAF
02402660	II	STGD1	*ABCA4*	50	24	Combination of changes in atrophic lesion size, best-corrected visual acuity (BCVA), and ocular assessments
01278277	I/II	STGD1	*ABCA4*	30	6	Focal electroretinogram
01505062	I/II	USH1B	*MYO7A*	18	12	Delay in retinal degeneration
02317887	I/IIa	XLRS	*RS1*	24	18	Visual function, ERG, OCT, visual field

AMD = age-related macular degeneration, BCVA = best-corrected visual acuity, CHM = choroideremia, CNV = choroidal neovascularization, ERG = electroretinography, FAF = fundus autofluorescence, LCA = Leber's congenital amaurosis, NCT = national clinical trial, OCT = optical coherence tomography, STGD1 = Stargardt disease, USH1B = Usher Syndrome Type IB, XLRS = X-linked juvenile retinoschisis.
