# Supplementary material for: Highly sensitive measurements of disease progression in rare disorders: Developing and validating a multimodal model of retinal degeneration in Stargardt disease
Source: PLoS One. 2017 Mar 29;12(3):e0174020. doi: 10.1371/journal.pone.0174020 (PMC5371327; doi:10.1371/journal.pone.0174020)
Supplement: S1 Appendix — (PDF) [file pone.0174020.s004.pdf]

```
/*
Syntax for "Weighting scores optimazation".
- Data first need to be transformed into changes from the baseline measurement in all
univariable biomarkers by retinal eccentricity (e).
- The macro is written for 5 biomarkers.
  1 = best-corrected visual acuity (delta_ecc_logmar)
  2 = questionably decreased autofluorescence (delta_ecc_qdaf)
  3 = definitely decreased autofluorescence (delta_ecc_ddaf)
  4 = transverse loss of the ellipsoid zone (delta_ecc_ez)
  5 = transverse loss of the external limiting membrane (delta_ecc_elm)
      composite (delta_ecc_comp)
- v: assigned weight by 5% steps
  w: relative weight of each biomarker
*/

options nonotes;
libname read 'location of the dataset';
data dataset;
set read.dataset;
run;
proc print data=dataset;
run;

/*
Start macro
*/

%macro weighting;
%let counter=1;
%do v1=0 %to 100 %by 5;
  %do v2=0 %to 100 %by 5;
    %do v3=0 %to 100 %by 5;
      %do v4=0 %to 100 %by 5;
        %do v5=0 %to 100 %by 5;
        %let sum = &v1 + &v2 + &v3 + &v4 + &v5;
        %if (&sum =100) %then %do;

data weighting;
set data;
    w1=.;
    w2=.;
    w3=.;
    w4=.;
    w5=.;
output;
run;

data weighting;
set weighting;
if (delta_ecc_logmar ne .) and (delta_ecc_qdaf ne .) and (delta_ecc_ddaf ne .) and (delta_ecc_ez
 ne .) and (delta_ecc_elm ne .) then do;
   w1 = &v1/(&v1 + &v2 +&v3 +&v4 +&v5);
   w2 = &v2/(&v1 + &v2 +&v3 +&v4 +&v5);
   w3 = &v3/(&v1 + &v2 +&v3 +&v4 +&v5);
   w4 = &v4/(&v1 + &v2 +&v3 +&v4 +&v5);
   w5 = &v5/(&v1 + &v2 +&v3 +&v4 +&v5);
   delta_ecc_comp = w1*delta_ecc_logmar + w2*delta_ecc_qdaf + w3*delta_ecc_ddaf +
   w4*delta_ecc_ez + w5*delta_ecc_elm;
end;
if (delta_ecc_logmar=.) and (delta_ecc_qdaf ne .) and (delta_ecc_ddaf ne .) and (delta_ecc_ez ne
 .) and (delta_ecc_elm ne .) then do;
   w2 = &v2/(&v2 +&v3 +&v4 +&v5);
   w3 = &v3/(&v2 +&v3 +&v4 +&v5);
   w4 = &v4/(&v2 +&v3 +&v4 +&v5);
   w5 = &v5/(&v2 +&v3 +&v4 +&v5);
```

```
   delta_ecc_comp = w2*delta_ecc_qdaf + w3*delta_ecc_ddaf + w4*delta_ecc_ez + w5*delta_ecc_elm;
end;
if (delta_ecc_logmar ne .) and (delta_ecc_qdaf = .) and (delta_ecc_ddaf ne .) and (delta_ecc_ez
ne .) and (delta_ecc_elm ne .) then do;
   w1 = &v1/(&v1 +&v3 +&v4 +&v5);
   w3 = &v3/(&v1 +&v3 +&v4 +&v5);
   w4 = &v4/(&v1 +&v3 +&v4 +&v5);
   w5 = &v5/(&v1 +&v3 +&v4 +&v5);
   delta_ecc_comp = w1*delta_ecc_logmar + w3*delta_ecc_ddaf + w4*delta_ecc_ez + w5*delta_ecc_elm;
end;
if (delta_ecc_logmar ne .) and (delta_ecc_qdaf ne .) and (delta_ecc_ddaf = .) and (delta_ecc_ez
ne .) and (delta_ecc_elm ne .) then do;
   w1 = &v1/(&v1 + &v2 +&v4 +&v5);
   w2 = &v2/(&v1 + &v2 +&v4 +&v5);
   w4 = &v4/(&v1 + &v2 +&v4 +&v5);
   w5 = &v5/(&v1 + &v2 +&v4 +&v5);
   delta_ecc_comp = w1*delta_ecc_logmar + w2*delta_ecc_qdaf + w4*delta_ecc_ez + w5*delta_ecc_elm;
end;
if (delta_ecc_logmar ne .) and (delta_ecc_qdaf ne .) and (delta_ecc_ddaf ne .) and (delta_ecc_ez
 = .) and (delta_ecc_elm ne .) then do;
   w1 = &v1/(&v1 + &v2 +&v3 +&v5);
   w2 = &v2/(&v1 + &v2 +&v3 +&v5);
   w3 = &v3/(&v1 + &v2 +&v3 +&v5);
   w5 = &v5/(&v1 + &v2 +&v3 +&v5);
   delta_ecc_comp = w1*delta_ecc_logmar + w2*delta_ecc_qdaf + w3*delta_ecc_ddaf +
   w5*delta_ecc_elm;
end;
if (delta_ecc_logmar ne .) and (delta_ecc_qdaf ne .) and (delta_ecc_ddaf ne .) and (delta_ecc_ez
 ne .) and (delta_ecc_elm = .) then do;
   w1 = &v1/(&v1 + &v2 +&v3 +&v4 );
   w2 = &v2/(&v1 + &v2 +&v3 +&v4 );
   w3 = &v3/(&v1 + &v2 +&v3 +&v4 );
   w4 = &v4/(&v1 + &v2 +&v3 +&v4 );
      delta_ecc_comp = w1*delta_ecc_logmar + w2*delta_ecc_qdaf + w3*delta_ecc_ddaf +
      w4*delta_ecc_ez ;
end;
if (delta_ecc_logmar=.) and (delta_ecc_qdaf = .) and (delta_ecc_ddaf ne .) and (delta_ecc_ez ne
.) and (delta_ecc_elm ne .) then do;
   w3 = &v3/(&v3 +&v4 +&v5);
   w4 = &v4/(&v3 +&v4 +&v5);
   w5 = &v5/(&v3 +&v4 +&v5);
   delta_ecc_comp = w3*delta_ecc_ddaf + w4*delta_ecc_ez + w5*delta_ecc_elm;
end;
if (delta_ecc_logmar=.) and (delta_ecc_qdaf ne .) and (delta_ecc_ddaf = .) and (delta_ecc_ez ne
.) and (delta_ecc_elm ne .) then do;
   w2 = &v2/(&v2 +&v4 +&v5);
   w4 = &v4/(&v2 +&v4 +&v5);
   w5 = &v5/(&v2 +&v4 +&v5);
   delta_ecc_comp = w2*delta_ecc_qdaf + w4*delta_ecc_ez + w5*delta_ecc_elm;
end;
if (delta_ecc_logmar=.) and (delta_ecc_qdaf ne .) and (delta_ecc_ddaf ne .) and (delta_ecc_ez =
.) and (delta_ecc_elm ne .) then do;
   w2 = &v2/(&v2 +&v3 +&v5);
   w3 = &v3/(&v2 +&v3 +&v5);
   w5 = &v5/(&v2 +&v3 +&v5);
   delta_ecc_comp = w2*delta_ecc_qdaf + w3*delta_ecc_ddaf  + w5*delta_ecc_elm;
end;
if (delta_ecc_logmar=.) and (delta_ecc_qdaf ne .) and (delta_ecc_ddaf ne .) and (delta_ecc_ez ne
 .) and (delta_ecc_elm = .) then do;
   w2 = &v2/(&v2 +&v3 +&v4 );
   w3 = &v3/(&v2 +&v3 +&v4 );
   w4 = &v4/(&v2 +&v3 +&v4 );
    delta_ecc_comp = w2*delta_ecc_qdaf + w3*delta_ecc_ddaf + w4*delta_ecc_ez;
end;
if (delta_ecc_logmar ne .) and (delta_ecc_qdaf = .) and (delta_ecc_ddaf = .) and (delta_ecc_ez
```

```
ne .) and (delta_ecc_elm ne .) then do;
   w1 = &v1/(&v1 +&v4 +&v5);
   w4 = &v4/(&v1 +&v4 +&v5);
   w5 = &v5/(&v1 +&v4 +&v5);
   delta_ecc_comp = w1*delta_ecc_logmar + w4*delta_ecc_ez + w5*delta_ecc_elm;
end;
if (delta_ecc_logmar ne .) and (delta_ecc_qdaf = .) and (delta_ecc_ddaf ne .) and (delta_ecc_ez
= .) and (delta_ecc_elm ne .) then do;
   w1 = &v1/(&v1 +&v3 +&v5);
   w3 = &v3/(&v1 +&v3 +&v5);
   w5 = &v5/(&v1 +&v3 +&v5);
   delta_ecc_comp = w1*delta_ecc_logmar + w3*delta_ecc_ddaf + w5*delta_ecc_elm;
end;
if (delta_ecc_logmar ne .) and (delta_ecc_qdaf = .) and (delta_ecc_ddaf ne .) and (delta_ecc_ez
ne .) and (delta_ecc_elm = .) then do;
   w1 = &v1/(&v1 +&v3 +&v4 );
   w3 = &v3/(&v1 +&v3 +&v4 );
   w4 = &v4/(&v1 +&v3 +&v4 );
   delta_ecc_comp = w1*delta_ecc_logmar + w3*delta_ecc_ddaf + w4*delta_ecc_ez;
end;
if (delta_ecc_logmar ne .) and (delta_ecc_qdaf ne .) and (delta_ecc_ddaf = .) and (delta_ecc_ez
= .) and (delta_ecc_elm ne .) then do;
   w1 = &v1/(&v1 + &v2 +&v5);
   w2 = &v2/(&v1 + &v2 +&v5);
   w5 = &v5/(&v1 + &v2 +&v5);
   delta_ecc_comp = w1*delta_ecc_logmar + w2*delta_ecc_qdaf +  w5*delta_ecc_elm;
end;
if (delta_ecc_logmar ne .) and (delta_ecc_qdaf ne .) and (delta_ecc_ddaf = .) and (delta_ecc_ez
ne .) and (delta_ecc_elm = .) then do;
   w1 = &v1/(&v1 + &v2 +&v4);
   w2 = &v2/(&v1 + &v2 +&v4);
   w4 = &v4/(&v1 + &v2 +&v4);
   delta_ecc_comp = w1*delta_ecc_logmar + w2*delta_ecc_qdaf + w4*delta_ecc_ez ;
end;
if (delta_ecc_logmar ne .) and (delta_ecc_qdaf ne .) and (delta_ecc_ddaf ne .) and (delta_ecc_ez
 = .) and (delta_ecc_elm = .) then do;
   w1 = &v1/(&v1 + &v2 +&v3 );
   w2 = &v2/(&v1 + &v2 +&v3 );
   w3 = &v3/(&v1 + &v2 +&v3 );
   delta_ecc_comp = w1*delta_ecc_logmar + w2*delta_ecc_qdaf + w3*delta_ecc_ddaf  ;
end;
if (delta_ecc_logmar ne .) and (delta_ecc_qdaf ne .) and (delta_ecc_ddaf = .) and (delta_ecc_ez
= .) and (delta_ecc_elm = .) then do;
   w1 = &v1/(&v1 + &v2 );
   w2 = &v2/(&v1 + &v2 );
   delta_ecc_comp = w1*delta_ecc_logmar + w2*delta_ecc_qdaf ;
end;
if (delta_ecc_logmar ne .) and (delta_ecc_qdaf = .) and (delta_ecc_ddaf ne .) and (delta_ecc_ez
= .) and (delta_ecc_elm = .) then do;
   w1 = &v1/(&v1 +&v3 );
   w3 = &v3/(&v1 +&v3 );
   delta_ecc_comp = w1*delta_ecc_logmar + w3*delta_ecc_ddaf;
end;
if (delta_ecc_logmar ne .) and (delta_ecc_qdaf = .) and (delta_ecc_ddaf = .) and (delta_ecc_ez
ne .) and (delta_ecc_elm = .) then do;
   w1 = &v1/(&v1 + &v4 );
   w4 = &v4/(&v1 + &v4 );
   delta_ecc_comp = w1*delta_ecc_logmar + w4*delta_ecc_ez;
end;
if (delta_ecc_logmar ne .) and (delta_ecc_qdaf = .) and (delta_ecc_ddaf = .) and (delta_ecc_ez =
 .) and (delta_ecc_elm ne .) then do;
   w1 = &v1/(&v1 + &v5);
   w5 = &v5/(&v1 + &v5);
   delta_ecc_comp = w1*delta_ecc_logmar + w5*delta_ecc_elm;
end;
```

```
if (delta_ecc_logmar =.) and (delta_ecc_qdaf ne .) and (delta_ecc_ddaf ne .) and (delta_ecc_ez =
 .) and (delta_ecc_elm = .) then do;
   w2 = &v2/( &v2 +&v3 );
   w3 = &v3/(&v2 +&v3 );
     delta_ecc_comp = w2*delta_ecc_qdaf + w3*delta_ecc_ddaf;
end;
if (delta_ecc_logmar =.) and (delta_ecc_qdaf ne .) and (delta_ecc_ddaf = .) and (delta_ecc_ez ne
 .) and (delta_ecc_elm = .) then do;
   w2 = &v2/( &v2 +&v4 );
   w4 = &v4/( &v2 +&v4 );
     delta_ecc_comp = w2*delta_ecc_qdaf  + w4*delta_ecc_ez ;
end;
if (delta_ecc_logmar =.) and (delta_ecc_qdaf ne .) and (delta_ecc_ddaf = .) and (delta_ecc_ez =
.) and (delta_ecc_elm ne .) then do;
   w2 = &v2/(&v2 +&v5);
   w5 = &v5/(&v2 +&v5);
   delta_ecc_comp = w2*delta_ecc_qdaf + w5*delta_ecc_elm;
end;
if (delta_ecc_logmar =.) and (delta_ecc_qdaf = .) and (delta_ecc_ddaf ne .) and (delta_ecc_ez ne
 .) and (delta_ecc_elm = .) then do;
   w3 = &v3/(&v3 +&v4 );
   w4 = &v4/(&v3 +&v4);
     delta_ecc_comp = w3*delta_ecc_ddaf + w4*delta_ecc_ez ;
end;
if (delta_ecc_logmar=.) and (delta_ecc_qdaf = .) and (delta_ecc_ddaf ne .) and (delta_ecc_ez =
.) and (delta_ecc_elm ne .) then do;
   w3 = &v3/(&v3 +&v5);
   w5 = &v5/(&v3 +&v5);
   delta_ecc_comp = w3*delta_ecc_ddaf +  w5*delta_ecc_elm;
end;
if (delta_ecc_logmar=.) and (delta_ecc_qdaf = .) and (delta_ecc_ddaf = .) and (delta_ecc_ez ne
.) and (delta_ecc_elm ne .) then do;
   w4 = &v4/(&v4 +&v5);
   w5 = &v5/(&v4 +&v5);
   delta_ecc_comp = w4*delta_ecc_ez + w5*delta_ecc_elm;
end;
if (delta_ecc_logmar ne .) and (delta_ecc_qdaf = .) and (delta_ecc_ddaf = .) and (delta_ecc_ez =
 .) and (delta_ecc_elm = .) then do;
   delta_ecc_comp = delta_ecc_logmar;
end;

if (delta_ecc_logmar =.) and (delta_ecc_qdaf ne .) and (delta_ecc_ddaf = .) and (delta_ecc_ez =
.) and (delta_ecc_elm = .) then do;
   delta_ecc_comp = delta_ecc_qdaf;
end;
if (delta_ecc_logmar =.) and (delta_ecc_qdaf = .) and (delta_ecc_ddaf ne .) and (delta_ecc_ez =
.) and (delta_ecc_elm = .) then do;
   delta_ecc_comp = delta_ecc_ddaf;
end;
if (delta_ecc_logmar =.) and (delta_ecc_qdaf = .) and (delta_ecc_ddaf = .) and (delta_ecc_ez ne
.) and (delta_ecc_elm = .) then do;
   delta_ecc_comp = delta_ecc_ez;
end;
if (delta_ecc_logmar =.) and (delta_ecc_qdaf = .) and (delta_ecc_ddaf = .) and (delta_ecc_ez =
.) and (delta_ecc_elm ne .) then do;
   delta_ecc_comp = delta_ecc_elm;
end;
output;
run;
proc datasets library=work;
   delete sf si sfi;
run;
ods select none;
proc mixed data=weighting method=reml maxiter=100000 covtest ;
Class patient_id eye;
```

```
Model delta_ecc_comp = delta_date_diff  / noint outpm=predmeans_std outp=predind_std solution;
Random delta_date_diff*patient_id delta_date_diff*patient_id(eye) /type=vc;
where delta_ecc_comp ne 0;
ods output solutionf=sf (rename=(estimate=slope)) covparms=si (rename=(estimate=residual));
run;
ods _all_ close;

   %if %sysfunc(exist(sf)) %then %do;

   data sf;
   set sf;
   record=1;
   keep record slope;
   run;

   data si;
   set si;
   record=1;
   if covparm='residual';
   keep record residual;
   run;

   data sfi;
   merge sf si; by record;
   msdr = slope/sqrt(residual);
   run;


   %if (&counter=1) %then %do;
      Data results_tot;
      set sfi;
      v1 = &v1; v2 = &v2; v3 = &v3; v4 = &v4; v5 = &v5;
      run;
   %end;
   %if (&counter>1) %then %do;
      Data results;
      set sfi;
      v1 = &v1; v2 = &v2; v3 = &v3; v4 = &v4; v5 = &v5;
      run;
      data results_tot;
      set results_tot results;
      run;
   %end;
   %let counter = &counter +1;

   %end;

        %end;
      %end;
      %end;
      %end;
      %end;
      %end;
%mend;
/*
End of macro
*/

%weighting;
run;

data read.results_tot;
set results_tot;
run;
```

```
ods output;
proc print data=read.results_tot;
run;
```
