# Supplementary material for: Highly sensitive measurements of disease progression in rare disorders: Developing and validating a multimodal model of retinal degeneration in Stargardt disease
Source: PLoS One. 2017 Mar 29;12(3):e0174020. doi: 10.1371/journal.pone.0174020 (PMC5371327; doi:10.1371/journal.pone.0174020)
Supplement: S2 Appendix — (PDF) [file pone.0174020.s005.pdf]

```
/*
Syntax for the trial simulations.
*/

options nonotes;
libname read 'location of the dataset';
data weighting;
set read.dataset;
/*
Set the weights for v1...v5.
*/
    v1=0;
    v2=25;
    v3=5;
    v4=55;
    v5=15;
    w1=.;
    w2=.;
    w3=.;
    w4=.;
    w5=.;
output;
run;

data weighting;
set weighting;
if (delta_ecc_logmar ne .) and (delta_ecc_qdaf ne .) and (delta_ecc_ddaf ne .) and (delta_ecc_ez
 ne .) and (delta_ecc_elm ne .) then do;
   w1 = v1/(v1 + v2 +v3 +v4 +v5);
   w2 = v2/(v1 + v2 +v3 +v4 +v5);
   w3 = v3/(v1 + v2 +v3 +v4 +v5);
   w4 = v4/(v1 + v2 +v3 +v4 +v5);
   w5 = v5/(v1 + v2 +v3 +v4 +v5);
   delta_ecc_comp = w1*delta_ecc_logmar + w2*delta_ecc_qdaf + w3*delta_ecc_ddaf +
   w4*delta_ecc_ez + w5*delta_ecc_elm;
end;
if (delta_ecc_logmar=.) and (delta_ecc_qdaf ne .) and (delta_ecc_ddaf ne .) and (delta_ecc_ez ne
 .) and (delta_ecc_elm ne .) then do;
   w2 = v2/(v2 +v3 +v4 +v5);
   w3 = v3/(v2 +v3 +v4 +v5);
   w4 = v4/(v2 +v3 +v4 +v5);
   w5 = v5/(v2 +v3 +v4 +v5);
   delta_ecc_comp = w2*delta_ecc_qdaf + w3*delta_ecc_ddaf + w4*delta_ecc_ez + w5*delta_ecc_elm;
end;
if (delta_ecc_logmar ne .) and (delta_ecc_qdaf = .) and (delta_ecc_ddaf ne .) and (delta_ecc_ez
ne .) and (delta_ecc_elm ne .) then do;
   w1 = v1/(v1 +v3 +v4 +v5);
   w3 = v3/(v1 +v3 +v4 +v5);
   w4 = v4/(v1 +v3 +v4 +v5);
   w5 = v5/(v1 +v3 +v4 +v5);
   delta_ecc_comp = w1*delta_ecc_logmar + w3*delta_ecc_ddaf + w4*delta_ecc_ez + w5*delta_ecc_elm;
end;
if (delta_ecc_logmar ne .) and (delta_ecc_qdaf ne .) and (delta_ecc_ddaf = .) and (delta_ecc_ez
ne .) and (delta_ecc_elm ne .) then do;
   w1 = v1/(v1 + v2 +v4 +v5);
   w2 = v2/(v1 + v2 +v4 +v5);
   w4 = v4/(v1 + v2 +v4 +v5);
   w5 = v5/(v1 + v2 +v4 +v5);
   delta_ecc_comp = w1*delta_ecc_logmar + w2*delta_ecc_qdaf + w4*delta_ecc_ez + w5*delta_ecc_elm;
end;
if (delta_ecc_logmar ne .) and (delta_ecc_qdaf ne .) and (delta_ecc_ddaf ne .) and (delta_ecc_ez
 = .) and (delta_ecc_elm ne .) then do;
   w1 = v1/(v1 + v2 +v3 +v5);
   w2 = v2/(v1 + v2 +v3 +v5);
   w3 = v3/(v1 + v2 +v3 +v5);
```

```
   w5 = v5/(v1 + v2 +v3 +v5);
   delta_ecc_comp = w1*delta_ecc_logmar + w2*delta_ecc_qdaf + w3*delta_ecc_ddaf +
   w5*delta_ecc_elm;
end;
if (delta_ecc_logmar ne .) and (delta_ecc_qdaf ne .) and (delta_ecc_ddaf ne .) and (delta_ecc_ez
 ne .) and (delta_ecc_elm = .) then do;
   w1 = v1/(v1 + v2 +v3 +v4 );
   w2 = v2/(v1 + v2 +v3 +v4 );
   w3 = v3/(v1 + v2 +v3 +v4 );
   w4 = v4/(v1 + v2 +v3 +v4 );
      delta_ecc_comp = w1*delta_ecc_logmar + w2*delta_ecc_qdaf + w3*delta_ecc_ddaf +
      w4*delta_ecc_ez ;
end;
if (delta_ecc_logmar=.) and (delta_ecc_qdaf = .) and (delta_ecc_ddaf ne .) and (delta_ecc_ez ne
.) and (delta_ecc_elm ne .) then do;
   w3 = v3/(v3 +v4 +v5);
   w4 = v4/(v3 +v4 +v5);
   w5 = v5/(v3 +v4 +v5);
   delta_ecc_comp = w3*delta_ecc_ddaf + w4*delta_ecc_ez + w5*delta_ecc_elm;
end;
if (delta_ecc_logmar=.) and (delta_ecc_qdaf ne .) and (delta_ecc_ddaf = .) and (delta_ecc_ez ne
.) and (delta_ecc_elm ne .) then do;
   w2 = v2/(v2 +v4 +v5);
   w4 = v4/(v2 +v4 +v5);
   w5 = v5/(v2 +v4 +v5);
   delta_ecc_comp = w2*delta_ecc_qdaf + w4*delta_ecc_ez + w5*delta_ecc_elm;
end;
if (delta_ecc_logmar=.) and (delta_ecc_qdaf ne .) and (delta_ecc_ddaf ne .) and (delta_ecc_ez =
.) and (delta_ecc_elm ne .) then do;
   w2 = v2/(v2 +v3 +v5);
   w3 = v3/(v2 +v3 +v5);
   w5 = v5/(v2 +v3 +v5);
   delta_ecc_comp = w2*delta_ecc_qdaf + w3*delta_ecc_ddaf  + w5*delta_ecc_elm;
end;
if (delta_ecc_logmar=.) and (delta_ecc_qdaf ne .) and (delta_ecc_ddaf ne .) and (delta_ecc_ez ne
 .) and (delta_ecc_elm = .) then do;
   w2 = v2/(v2 +v3 +v4 );
   w3 = v3/(v2 +v3 +v4 );
   w4 = v4/(v2 +v3 +v4 );
    delta_ecc_comp = w2*delta_ecc_qdaf + w3*delta_ecc_ddaf + w4*delta_ecc_ez;
end;
if (delta_ecc_logmar ne .) and (delta_ecc_qdaf = .) and (delta_ecc_ddaf = .) and (delta_ecc_ez
ne .) and (delta_ecc_elm ne .) then do;
   w1 = v1/(v1 +v4 +v5);
   w4 = v4/(v1 +v4 +v5);
   w5 = v5/(v1 +v4 +v5);
   delta_ecc_comp = w1*delta_ecc_logmar + w4*delta_ecc_ez + w5*delta_ecc_elm;
end;
if (delta_ecc_logmar ne .) and (delta_ecc_qdaf = .) and (delta_ecc_ddaf ne .) and (delta_ecc_ez
= .) and (delta_ecc_elm ne .) then do;
   w1 = v1/(v1 +v3 +v5);
   w3 = v3/(v1 +v3 +v5);
   w5 = v5/(v1 +v3 +v5);
   delta_ecc_comp = w1*delta_ecc_logmar + w3*delta_ecc_ddaf + w5*delta_ecc_elm;
end;
if (delta_ecc_logmar ne .) and (delta_ecc_qdaf = .) and (delta_ecc_ddaf ne .) and (delta_ecc_ez
ne .) and (delta_ecc_elm = .) then do;
   w1 = v1/(v1 +v3 +v4 );
   w3 = v3/(v1 +v3 +v4 );
   w4 = v4/(v1 +v3 +v4 );
   delta_ecc_comp = w1*delta_ecc_logmar + w3*delta_ecc_ddaf + w4*delta_ecc_ez;
end;
if (delta_ecc_logmar ne .) and (delta_ecc_qdaf ne .) and (delta_ecc_ddaf = .) and (delta_ecc_ez
= .) and (delta_ecc_elm ne .) then do;
   w1 = v1/(v1 + v2 +v5);
```

```
   w2 = v2/(v1 + v2 +v5);
   w5 = v5/(v1 + v2 +v5);
   delta_ecc_comp = w1*delta_ecc_logmar + w2*delta_ecc_qdaf +  w5*delta_ecc_elm;
end;
if (delta_ecc_logmar ne .) and (delta_ecc_qdaf ne .) and (delta_ecc_ddaf = .) and (delta_ecc_ez
ne .) and (delta_ecc_elm = .) then do;
   w1 = v1/(v1 + v2 +v4);
   w2 = v2/(v1 + v2 +v4);
   w4 = v4/(v1 + v2 +v4);
   delta_ecc_comp = w1*delta_ecc_logmar + w2*delta_ecc_qdaf + w4*delta_ecc_ez ;
end;
if (delta_ecc_logmar ne .) and (delta_ecc_qdaf ne .) and (delta_ecc_ddaf ne .) and (delta_ecc_ez
 = .) and (delta_ecc_elm = .) then do;
   w1 = v1/(v1 + v2 +v3 );
   w2 = v2/(v1 + v2 +v3 );
   w3 = v3/(v1 + v2 +v3 );
   delta_ecc_comp = w1*delta_ecc_logmar + w2*delta_ecc_qdaf + w3*delta_ecc_ddaf  ;
end;
if (delta_ecc_logmar ne .) and (delta_ecc_qdaf ne .) and (delta_ecc_ddaf = .) and (delta_ecc_ez
= .) and (delta_ecc_elm = .) then do;
   w1 = v1/(v1 + v2 );
   w2 = v2/(v1 + v2 );
   delta_ecc_comp = w1*delta_ecc_logmar + w2*delta_ecc_qdaf ;
end;
if (delta_ecc_logmar ne .) and (delta_ecc_qdaf = .) and (delta_ecc_ddaf ne .) and (delta_ecc_ez
= .) and (delta_ecc_elm = .) then do;
   w1 = v1/(v1 +v3 );
   w3 = v3/(v1 +v3 );
   delta_ecc_comp = w1*delta_ecc_logmar + w3*delta_ecc_ddaf;
end;
if (delta_ecc_logmar ne .) and (delta_ecc_qdaf = .) and (delta_ecc_ddaf = .) and (delta_ecc_ez
ne .) and (delta_ecc_elm = .) then do;
   w1 = v1/(v1 + v4 );
   w4 = v4/(v1 + v4 );
   delta_ecc_comp = w1*delta_ecc_logmar + w4*delta_ecc_ez;
end;
if (delta_ecc_logmar ne .) and (delta_ecc_qdaf = .) and (delta_ecc_ddaf = .) and (delta_ecc_ez =
 .) and (delta_ecc_elm ne .) then do;
   w1 = v1/(v1 + v5);
   w5 = v5/(v1 + v5);
   delta_ecc_comp = w1*delta_ecc_logmar + w5*delta_ecc_elm;
end;
if (delta_ecc_logmar =.) and (delta_ecc_qdaf ne .) and (delta_ecc_ddaf ne .) and (delta_ecc_ez =
 .) and (delta_ecc_elm = .) then do;
   w2 = v2/( v2 +v3 );
   w3 = v3/(v2 +v3 );
     delta_ecc_comp = w2*delta_ecc_qdaf + w3*delta_ecc_ddaf;
end;
if (delta_ecc_logmar =.) and (delta_ecc_qdaf ne .) and (delta_ecc_ddaf = .) and (delta_ecc_ez ne
 .) and (delta_ecc_elm = .) then do;
   w2 = v2/( v2 +v4 );
   w4 = v4/( v2 +v4 );
     delta_ecc_comp = w2*delta_ecc_qdaf  + w4*delta_ecc_ez ;
end;
if (delta_ecc_logmar =.) and (delta_ecc_qdaf ne .) and (delta_ecc_ddaf = .) and (delta_ecc_ez =
.) and (delta_ecc_elm ne .) then do;
   w2 = v2/(v2 +v5);
   w5 = v5/(v2 +v5);
   delta_ecc_comp = w2*delta_ecc_qdaf + w5*delta_ecc_elm;
end;
if (delta_ecc_logmar =.) and (delta_ecc_qdaf = .) and (delta_ecc_ddaf ne .) and (delta_ecc_ez ne
 .) and (delta_ecc_elm = .) then do;
   w3 = v3/(v3 +v4 );
   w4 = v4/(v3 +v4);
     delta_ecc_comp = w3*delta_ecc_ddaf + w4*delta_ecc_ez ;
```

```
end;
if (delta_ecc_logmar=.) and (delta_ecc_qdaf = .) and (delta_ecc_ddaf ne .) and (delta_ecc_ez =
.) and (delta_ecc_elm ne .) then do;
   w3 = v3/(v3 +v5);
   w5 = v5/(v3 +v5);
   delta_ecc_comp = w3*delta_ecc_ddaf +  w5*delta_ecc_elm;
end;
if (delta_ecc_logmar=.) and (delta_ecc_qdaf = .) and (delta_ecc_ddaf = .) and (delta_ecc_ez ne
.) and (delta_ecc_elm ne .) then do;
   w4 = v4/(v4 +v5);
   w5 = v5/(v4 +v5);
   delta_ecc_comp = w4*delta_ecc_ez + w5*delta_ecc_elm;
end;
if (delta_ecc_logmar ne .) and (delta_ecc_qdaf = .) and (delta_ecc_ddaf = .) and (delta_ecc_ez =
 .) and (delta_ecc_elm = .) then do;
   delta_ecc_comp = delta_ecc_logmar;
end;

if (delta_ecc_logmar =.) and (delta_ecc_qdaf ne .) and (delta_ecc_ddaf = .) and (delta_ecc_ez =
.) and (delta_ecc_elm = .) then do;
   delta_ecc_comp = delta_ecc_qdaf;
end;

if (delta_ecc_logmar =.) and (delta_ecc_qdaf = .) and (delta_ecc_ddaf ne .) and (delta_ecc_ez =
.) and (delta_ecc_elm = .) then do;
   delta_ecc_comp = delta_ecc_ddaf;
end;

if (delta_ecc_logmar =.) and (delta_ecc_qdaf = .) and (delta_ecc_ddaf = .) and (delta_ecc_ez ne
.) and (delta_ecc_elm = .) then do;
   delta_ecc_comp = delta_ecc_ez;
end;

if (delta_ecc_logmar =.) and (delta_ecc_qdaf = .) and (delta_ecc_ddaf = .) and (delta_ecc_ez =
.) and (delta_ecc_elm ne .) then do;
   delta_ecc_comp = delta_ecc_elm;
end;
output;
run;

data weighting;
set weighting;
if (measurements - measurement > 1) then do;
   output;
end;
if (measurement+1 = measurements) then do;
   output;
   measurement = measurement+1;
   delta_date_diff = delta_date_diff + 1;
   delta_ecc_logmar=.;
   delta_ecc_qdaf=.;
   delta_ecc_ddaf=.;
   delta_ecc_ez=.;
   delta_ecc_elm=.;
   delta_ecc_comp=.;
      output;
   measurement = measurement+1;
   delta_date_diff = delta_date_diff + 1;
   delta_ecc_logmar=.;
   delta_ecc_qdaf=.;
   delta_ecc_ddaf=.;
   delta_ecc_ez=.;
   delta_ecc_elm=.;
   delta_ecc_comp=.;
   output;
```

```
end;

run;


proc mixed data=weighting method=reml covtest;
Class patient_id eye;
Model delta_ecc_comp = delta_date_diff  / noint outpm=predmeans_std outp=predind_std solution;
Random delta_date_diff /type=vc subject=patient_id;
Random delta_date_diff /type=vc subject=patient_id*eye;
where delta_ecc_comp ne 0;
run;

data predind_std;
set predind_std;
variance =resid**2;
dummy=1;
run;
proc print data=predind_std;
run;
proc means data=predind_std;
var variance;
output out=test mean=mean_variance;
where delta_date_diff ne 0;
run;

data test;
set test;
residual = sqrt(mean_variance);
dummy=1;
run;

data predind2_std;
merge predind_std test; by dummy;
measurement_new = measurement - (measurements-2);
if ((measurement - measurements) ge -1);
keep patient_id eye pred residual measurement_new delta_ecc_comp;
run;

proc print data=predind2_std;
run;

data one;
set predind2_std;
if measurement_new=1 and eye='OD';
rename delta_ecc_comp=delta_ecc_comp_OD_known;
rename pred=pred_OD_0year;
run;
data one; set one;
keep patient_id residual delta_ecc_comp_OD_known pred_OD_0year;
run;
data two;
set predind2_std;
if measurement_new=1 and eye='OS';
rename delta_ecc_comp=delta_ecc_comp_OS_known;
rename pred=pred_OS_0year;
run;
data two; set two;
keep patient_id delta_ecc_comp_OS_known pred_OS_0year;
run;
data three;
set predind2_std;
if measurement_new=2 and eye='OD';
rename pred=pred_OD_1year;
run;
```

```
data three; set three;
keep patient_id pred_OD_1year;
run;
data four;
set predind2_std;
if measurement_new=2 and eye='OS';
rename pred=pred_OS_1year;
run;
data four; set four;
keep patient_id pred_OS_1year;
run;
data five;
set predind2_std;
if measurement_new=3 and eye='OD';
rename pred=pred_OD_2year;
run;
data five; set five;
keep patient_id pred_OD_2year;
run;
data six;
set predind2_std;
if measurement_new=3 and eye='OS';
rename pred=pred_OS_2year;
run;
data six; set six;
keep patient_id pred_OS_2year;
run;

data patient;
merge one two three four five six; by patient_id;
run;

proc print data=patient;
run;


/*
Start macro: treat random eye. Follow-up: 2 years.
*/

%macro simulate(effect);

proc datasets library=work;
   delete results_tot;
run;

%do k=1 %to 10000;
   data patient2;
   set patient;

random_treat_eye = rand('Bernouilli', 0.5);
if (random_treat_eye = 0) then do;
 sim_delta_ecc_untreated_baseline =  delta_ecc_comp_OS_known;
 sim_delta_ecc_untreated_2year = rand('normal',pred_OS_2year,residual);
  if (&effect=0) then   effect_size = 0;
 else do until (effect_size => rand('uniform') AND effect_size =< (&effect/100)*rand('uniform'));
  effect_size = rand ('normal',&effect/100,0.10);
  end;
effect_2year =  ( pred_OD_2year - pred_OD_0year )* effect_size;
 sim_delta_ecc_treated_baseline = delta_ecc_comp_OD_known;
 sim_delta_ecc_treated_2year = rand('normal',pred_OD_2year - effect_2year,residual);
end;

if (random_treat_eye = 1) then do;
 sim_delta_ecc_untreated_baseline =  delta_ecc_comp_OD_known;
```

```
 sim_delta_ecc_untreated_2year = rand('normal',pred_OD_2year,residual);
  if (&effect=0) then   effect_size = 0;
 else do until (effect_size => rand('uniform') AND effect_size =< (&effect/100)*rand('uniform'));
  effect_size = rand ('normal',&effect/100,0.10);
  end;
effect_2year =  ( pred_OS_2year - pred_OS_0year )* effect_size;
 sim_delta_ecc_treated_baseline = delta_ecc_comp_OS_known;
 sim_delta_ecc_treated_2year = rand('normal',pred_OS_2year - effect_2year,residual);
end;

 prog_ecc_untreated_2year =     sim_delta_ecc_untreated_2year - sim_delta_ecc_untreated_baseline;
 prog_ecc_treated_2year =   sim_delta_ecc_treated_2year - sim_delta_ecc_treated_baseline;
 delta_prog_2year = prog_ecc_untreated_2year -  prog_ecc_treated_2year;

run;

   proc means data=patient2 noprint;
   var delta_prog_2year;
   output out=results stderr=stderr mean=mean n=n;
   run;

   %if (&k=1) %then %do;
      Data results_tot;
      set results;
      simulation=&k;
      t=abs(mean/stderr);
      p = 2*(1 - cdf('T', t, n-1));
      run;
   %end;
   %if (&k>1) %then %do;
      Data results;
      set results;
      simulation=&k;
      t=abs(mean/stderr);
      p = 2*(1 - cdf('T', t, n-1));
      run;
      data results_tot;
      set results_tot results;
      run;
   %end;
%end;

Title 'Reduction' &effect  '(%)';
proc univariate data=results_tot;
var p mean;
histogram p mean;
run;

data results_tot;
set results_tot;
if (P<0.05 AND mean>0) then reject=1;
else reject=0;
run;

proc freq data=results_tot;
tables reject;
run;
%mend;


/*
Start macro: treat random eye. Follow-up: 1 year.
*/

%macro simulate(effect);
```

```
proc datasets library=work;
   delete results_tot;
run;

%do k=1 %to 10000;
   data patient2;
   set patient;

random_treat_eye = rand('Bernouilli', 0.5);
if (random_treat_eye = 0) then do;
 sim_delta_ecc_untreated_baseline =  delta_ecc_comp_OS_known;
 sim_delta_ecc_untreated_1year = rand('normal',pred_OS_1year,residual);
 if (&effect=0) then   effect_size = 0;
 else do until (effect_size => rand('uniform') AND effect_size =< (&effect/100)*rand('uniform'));
  effect_size = rand ('normal',&effect/100,0.10);
 end;

effect_1year =  ( pred_OD_1year - pred_OD_0year )* effect_size;
 sim_delta_ecc_treated_baseline = delta_ecc_comp_OD_known;
 sim_delta_ecc_treated_1year = rand('normal',pred_OD_1year - effect_1year,residual);
end;

if (random_treat_eye = 1) then do;
 sim_delta_ecc_untreated_baseline =  delta_ecc_comp_OD_known;
 sim_delta_ecc_untreated_1year = rand('normal',pred_OD_1year,residual);
  if (&effect=0) then   effect_size = 0;
 else do until (effect_size => rand('uniform') AND effect_size =< (&effect/100)*rand('uniform'));
  effect_size = rand ('normal',&effect/100,0.10);
  end;
 effect_1year =  ( pred_OS_1year - pred_OS_0year )* effect_size;
 sim_delta_ecc_treated_baseline = delta_ecc_comp_OS_known;
 sim_delta_ecc_treated_1year = rand('normal',pred_OS_1year - effect_1year,residual);
end;

 prog_ecc_untreated_1year =     sim_delta_ecc_untreated_1year - sim_delta_ecc_untreated_baseline;
 prog_ecc_treated_1year =   sim_delta_ecc_treated_1year - sim_delta_ecc_treated_baseline;
 delta_prog_1year = prog_ecc_untreated_1year -  prog_ecc_treated_1year;

run;

   proc means data=patient2 noprint;
   var delta_prog_1year;
   output out=results stderr=stderr mean=mean n=n;
   run;

   %if (&k=1) %then %do;
      Data results_tot;
      set results;
      simulation=&k;
      t=abs(mean/stderr);
      p = 2*(1 - cdf('T', t, n-1));
      run;
   %end;
   %if (&k>1) %then %do;
      Data results;
      set results;
      simulation=&k;
      t=abs(mean/stderr);
      p = 2*(1 - cdf('T', t, n-1));
      run;
      data results_tot;
      set results_tot results;
      run;
   %end;
```

```
%end;

Title 'Reduction' &effect  '(%)';
proc univariate data=results_tot;
var p mean;
histogram p mean;
run;

data results_tot;
set results_tot;
if (P<0.05 AND mean>0) then reject=1;
else reject=0;
run;

proc freq data=results_tot;
tables reject;
run;
%mend;


/*
Start macro: treat worst eye. Follow-up: 2 years.
*/

%macro simulate(effect);

proc datasets library=work;
   delete results_tot;
run;

%do k=1 %to 10000;
   data patient2;
   set patient;

treat_worst_eye = 0;
if (delta_ecc_comp_OS_known > delta_ecc_comp_OD_known) then treat_worst_eye = 1;
if (treat_worst_eye = 0) then do;
 sim_delta_ecc_untreated_baseline =  delta_ecc_comp_OS_known;
 sim_delta_ecc_untreated_2year = rand('normal',pred_OS_2year,residual);

 if (&effect=0) then   effect_size = 0;
 else do until (effect_size => rand('uniform') AND effect_size =< (&effect/100)*rand('uniform'));
  effect_size = rand ('normal',&effect/100,0.10);
  end;

 effect_2year =  ( pred_OD_2year - pred_OD_0year )* effect_size;
 sim_delta_ecc_treated_baseline = delta_ecc_comp_OD_known;
 sim_delta_ecc_treated_2year = rand('normal',pred_OD_2year - effect_2year,residual);
end;

if (treat_worst_eye = 1) then do;
 sim_delta_ecc_untreated_baseline =  delta_ecc_comp_OD_known;
 sim_delta_ecc_untreated_2year = rand('normal',pred_OD_2year,residual);

 if (&effect=0) then   effect_size = 0;
 else do until (effect_size => rand('uniform') AND effect_size =< (&effect/100)*rand('uniform'));
  effect_size = rand ('normal',&effect/100,0.10);
  end;
 effect_2year =  ( pred_OS_2year - pred_OS_0year )* effect_size;
 sim_delta_ecc_treated_baseline = delta_ecc_comp_OS_known;
 sim_delta_ecc_treated_2year = rand('normal',pred_OS_2year - effect_2year,residual);
end;

 prog_ecc_untreated_2year =     sim_delta_ecc_untreated_2year - sim_delta_ecc_untreated_baseline;
 prog_ecc_treated_2year =   sim_delta_ecc_treated_2year - sim_delta_ecc_treated_baseline;
```

```
 delta_prog_2year = prog_ecc_untreated_2year -  prog_ecc_treated_2year;

run;

   proc means data=patient2 noprint;
   var delta_prog_2year;
   output out=results stderr=stderr mean=mean n=n;
   run;

   %if (&k=1) %then %do;
      Data results_tot;
      set results;
      simulation=&k;
      t=abs(mean/stderr);
      p = 2*(1 - cdf('T', t, n-1));
      run;
   %end;
   %if (&k>1) %then %do;
      Data results;
      set results;
      simulation=&k;
      t=abs(mean/stderr);
      p = 2*(1 - cdf('T', t, n-1));
      run;
      data results_tot;
      set results_tot results;
      run;
   %end;
%end;

Title 'Reduction: ' &effect  '(%)';
proc univariate data=results_tot;
var p mean;
histogram p mean;
run;

data results_tot;
set results_tot;
if (P<0.05 AND mean>0) then reject=1;
else reject=0;
run;

proc freq data=results_tot;
tables reject;
run;
%mend;


/*
Simulate unpaired trial, random eye treated. Follow-up: 2 years.
*/

%macro simulate(effect);

proc datasets library=work;
   delete results_tot;
run;

%do k=1 %to 10000;
      proc surveyselect noprint data=patient out=split samprate=.5 outall;
      run;
      data treated_patients untreated_patients;
      set split;
      if selected = 1 then
      output treated_patients;
```

```
      else output untreated_patients;
      run;

data untreated_patients;
set untreated_patients;

treated=0;
sim_delta_ecc_OS_baseline = delta_ecc_comp_OS_known;
sim_delta_ecc_OS_2year = rand('normal',pred_OS_2year,residual);
sim_delta_ecc_OD_baseline = delta_ecc_comp_OD_known;
sim_delta_ecc_OD_2year = rand('normal',pred_OD_2year,residual);
run;

data treated_patients;
set treated_patients;

treated=1;

 if (&effect=0) then   effect_size = 0;
 else do until (effect_size => rand('uniform') AND effect_size =< (&effect/100)*rand('uniform'));
  effect_size = rand ('normal',&effect/100,0.10);
  end;
effect_2year =  ( pred_OD_2year - pred_OD_0year )* effect_size;
sim_delta_ecc_OD_baseline = delta_ecc_comp_OD_known;
sim_delta_ecc_OD_2year = rand('normal',pred_OD_2year - effect_2year,residual);
effect_2year =  ( pred_OS_2year - pred_OS_0year )* effect_size;
sim_delta_ecc_OS_baseline = delta_ecc_comp_OS_known;
sim_delta_ecc_OS_2year = rand('normal',pred_OS_2year - effect_2year,residual);
run;

data patient2;
set untreated_patients treated_patients;
 prog_ecc_OD_2year =    sim_delta_ecc_OD_2year - sim_delta_ecc_OD_baseline;
 prog_ecc_OS_2year =    sim_delta_ecc_OS_2year - sim_delta_ecc_OS_baseline;
 prog_ecc_ODS_2year = mean(prog_ecc_OD_2year,prog_ecc_OS_2year);

run;

   proc ttest data=patient2;
   class treated;
   var prog_ecc_ODS_2year;
   ods output Ttests=ttest_output Statistics=stats;
   run;

   data stats1;
   set stats;
   if class='0';
   id=1;
   rename mean=mean_untreated;
   run;
   data stats1;
   set stats1;
   keep id mean_untreated;
   run;
   data stats2;
   set stats;
   if class='1';
   id=1;
   rename mean=mean_treated;
   run;
   data stats2;
   set stats2;
   keep id mean_treated;
   run;
   data ttest2;
```

```
   set ttest_output;
   if method='Pooled';
   id=1;
   keep id probt;
   run;
   data results;
   merge stats1 stats2 ttest2;
   difference = mean_untreated - mean_treated;
   reject=0;
   if (difference>0) and (probt < 0.05) then do;
     reject=1;
   end;
   by id;
   drop id;
   run;

   %if (&k=1) %then %do;
      Data results_tot;
      set results;
      simulation=&k;
      run;
   %end;
   %if (&k>1) %then %do;
      Data results;
      set results;
      simulation=&k;
      run;
      data results_tot;
      set results_tot results;
      run;
   %end;
%end;

Title 'Reduction' &effect  '(%)';
proc univariate data=results_tot;
var probt difference;
histogram probt difference;
run;

proc freq data=results_tot;
tables reject;
run;
%mend;

%simulate(0);
%simulate(5);
%simulate(10);
%simulate(15);
%simulate(20);
%simulate(25);
%simulate(30);
%simulate(35);
%simulate(40);
%simulate(45);
%simulate(50);
%simulate(55);
%simulate(60);
%simulate(65);
%simulate(70);
%simulate(75);
%simulate(80);
%simulate(85);
%simulate(90);
%simulate(95);
%simulate(100);
```
